# Bleeding assessment tools and quality of life in bleeding disorder of unknown cause

**DOI:** 10.1016/j.rpth.2026.103362

**Published:** 2026-01-23

**Authors:** Dino Mehic, Johanna Gebhart

**Affiliations:** Division of Hematology and Hemostaseology, Department of Medicine I, Medical University of Vienna, Vienna, Austria

Although bleeding symptoms are common in the general population [1], they are clinically meaningful in a proportion of individuals in whom they indicate an underlying bleeding disorder [2]. Therefore, objective recording of the bleeding phenotype is relevant to possibly identify individuals at risk for bleeding and prompt hemostatic investigations. However, differentiating patients with clinically meaningful bleeding from those with trivial symptoms is challenging, even for experienced hemostasis specialists, and requires careful integration of an individual and familial bleeding history and physical examination [3,4] The majority of patients presenting to tertiary care centers with mild-to-moderate bleeding symptoms are ultimately classified as having a bleeding disorder of unknown cause (BDUC). In such patients, the bleeding phenotype is the only detectable manifestation of disease, with standard coagulation and platelet assays remaining normal (Figure) [5]. Together with the reliance on bleeding history to establish a diagnosis of von Willebrand disease in patients with von Willebrand factor levels of 30 to 50 IU/dL [6], this underscores the central role of rigorous clinical phenotyping in the evaluation of patients with a suspected bleeding tendency.

**Figure:**
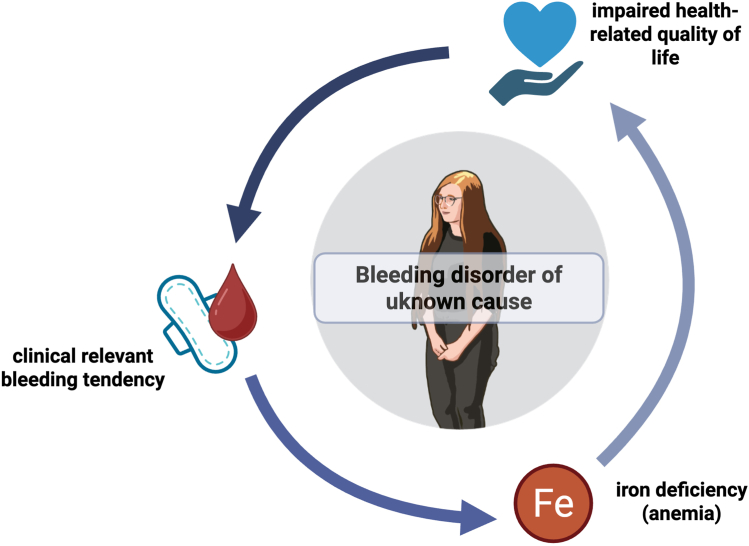
Interplay between bleeding tendency, iron deficiency, and quality of life in bleeding disorder of unknown cause.

Bleeding assessment tools (BATs) were developed to standardize and quantify lifelong bleeding symptoms, particularly in the evaluation of suspected inherited bleeding disorders [7]. While structured interviews to gather the bleeding phenotype have been shown to perform well as screening tools in primary care settings [8], the clinical utility of BATs to differentiate between patients with bleeding disorders may remain limited in tertiary care settings [9]. In this context, important limitations of BATs must be recognized: as BAT scores are cumulative, they are less informative in individuals with limited hemostatic challenges (eg, no surgeries or childbirth in patient history). Scores increase with age, yet age-adjusted thresholds are not available or routinely applied, and bleeding events occurring under treatment or prophylaxis are not consistently captured [10]. In addition, BATs primarily reflect bleeding severity rather than bleeding frequency. Despite these limitations, the International Society on Thrombosis and Haemostasis (ISTH)-BAT has been incorporated into the diagnostic evaluation of mild bleeding disorders and BDUC to guide clinical workup [4]. However, its routine use in practice is constrained by the time required for administration and the need for trained personnel. To mitigate these barriers, a self-administered version of the ISTH-BAT (Self-BAT) was developed, enabling patients to report bleeding symptoms independently [11]. Nonetheless, the performance and clinical utility of the Self-BAT in patients undergoing evaluation for BDUC has not been well studied.

While BATs have traditionally been used to standardize bleeding history and support diagnostic decision making [9], they may also function as integrated measures of bleeding burden [7], capturing dimensions of disease that extend beyond laboratory-defined pathology. Although impaired health-related quality of life (HrQoL) has been described in patients with mild bleeding disorders and BDUC [12,13], the relationship between bleeding severity, specific bleeding manifestations, and patient-reported outcomes has remained incompletely characterized.

In this issue of *Research and Practice in Thrombosis and Haemostasis*, Berkowitz et al. [14] report data on the bleeding phenotype and quality of life from 101 patients with bleeding symptoms referred to a hemostatic investigation in a tertiary care center. Of these patients, 21% were categorized as not having a relevant bleeding tendency by a hemostasis expert, and 46% were diagnosed with BDUC. This is consistent with reports from diverse geographic regions, in which approximately 40% to 70% of patients with a clinically relevant bleeding tendency investigated at tertiary care centers ultimately receive a diagnosis of BDUC [3]. The presented cohort demonstrated strong concordance on clinical features with previously described mild-to-moderate bleeding disorder populations [3]. Iron deficiency was common in this study, affecting 42.9% of women, mirroring observations from the Vienna Bleeding Biobank and reinforcing the close link between chronic bleeding symptoms and iron depletion in this population [15]. In the current study, the total Self-BAT and ISTH-BAT scores showed good overall correlation, particularly for bleeding domains related to postpartum hemorrhage and menstrual bleeding [14]. Nevertheless, clinically relevant discrepancies emerged for distinct domains. Patients reported higher scores for hematuria, gastrointestinal bleeding, and muscle bleeding on the Self-BAT, whereas clinician-administered ISTH-BAT scores were higher for bleeding associated with tooth extraction. These differences likely reflect variations in symptom interpretation between patients and clinicians, recall bias, differences in perceived severity, and the dynamics of a structured clinical interview, as the authors discuss in their study publication. Both the ISTH-BAT and the Self-BAT were abnormal in approximately 70% of patients using sex-specific cutoffs. However, this contrasts with findings from a Danish cohort of 465 patients evaluated for bleeding symptoms, in which 75% were classified as having BDUC and nearly all patients had abnormal Self-BAT scores (95% of women with scores ≥6 and 98% of men with scores ≥4) [11].

Patients with bleeding disorders commonly experience impairments in HrQoL, a finding well established for conditions such as hemophilia and von Willebrand disease [12,16]. In contrast, data in patients with BDUC have been more limited, and the determinants of impaired HrQoL in this population remain incompletely understood [13]. Although bleeding severity would intuitively be expected to contribute to HrQoL impairment, two independent cohorts of patients with BDUC failed to demonstrate a clear association between bleeding phenotype or bleeding severity and HrQoL impairment [13,17]. These observations suggest that factors beyond bleeding frequency alone, potentially including chronic symptom burden, iron deficiency, pain, or psychosocial stressors may substantially shape patient-reported outcomes in BDUC. In this context, the study by Berkowitz et al. [14] provides important new insight by demonstrating, for the first time, that higher scores on both clinician-administered and patient-reported BATs are associated with impairments across multiple quality-of-life domains in patients with BDUC as well as other bleeding disorders. Higher ISTH-BAT scores were associated with greater HrQoL impairment as measured by Patient-Reported Outcomes Measurement Information System (PROMIS) domains, and similar associations were observed for the Self-BAT, independent of age and sex. As mentioned earlier, these findings contrast with prior analyses from the Vienna Bleeding Biobank and a Dutch cohort using the Short Form (SF)-36 questionnaire, which did not identify similar strong associations. The differences that may plausibly be explained by variation in assessment timing, cohort composition, and the higher proportion of patients with more severe bleeding phenotypes in the present study.

In summary, Berkowitz et al. [14] demonstrated that patient-reported bleeding assessment using the Self-BAT correlates well with clinician-administered ISTH-BAT scores, supporting its validity as a pragmatic screening tool to identify individuals with clinically relevant bleeding. This is particularly important in routine practice, where scalable instruments are needed to distinguish true bleeding phenotypes from trivial bleeding symptoms. Importantly, the association of both BATs with impairments across multiple HRQoL domains reframes BATs as measures of disease burden rather than diagnostic instruments alone. These findings move the field beyond diagnostic classification toward a patient-centered framework in which bleeding severity informs clinical impact and care needs. Future efforts should integrate BATs with patient-reported outcomes, laboratory phenotyping, and biological modifiers to guide not only diagnosis but also the intensity and structure of clinical management. Repositioning BATs to address not only who bleeds but who suffers and how much represents a critical step toward improving care for patients with BDUC and other mild bleeding disorders.
